# Sleep-Disordered Breathing and Idiopathic Normal-Pressure Hydrocephalus: Recent Pathophysiological Advances

**DOI:** 10.1007/s11910-019-0952-9

**Published:** 2019-05-29

**Authors:** Gustavo C. Román, Robert E. Jackson, Steve H. Fung, Y. Jonathan Zhang, Aparajitha K. Verma

**Affiliations:** 1 Department of Neurology, Methodist Neurological Institute and Houston Methodist Hospital Research Institute for Academic Medicine, Houston, TX USA; 2 Department of Neurology, Weill Cornell Medical College, Cornell University, New York, NY USA; 3 Department of Medicine, Houston Methodist Hospital and Houston Research Institute for Academic Medicine, Houston, TX USA; 4 Department of Medicine, Weill Cornell Medical College, Cornell University, New York, NY USA; 5 Department of Radiology MRI Core, Houston Methodist Hospital and Methodist Research Institute for Academic Medicine, Houston, TX USA; 6 Department of Radiology Weill Cornell Medical College, Cornell University, New York, NY USA; 7 Department of Neurosurgery, Methodist Neurological Institute and Houston Methodist Hospital Research Institute for Academic Medicine, Houston, TX USA; 8 Department of Neurosurgery, Weill Cornell Medical College, Cornell University, New York, NY USA; 9 Sleep Laboratory Houston Methodist Hospital, Houston, TX USA

**Keywords:** Cerebral venous circulation, Glymphatic system, Normal-pressure hydrocephalus, Obstructive sleep apnea, Sleep-disordered breathing, Vascular risk factors

## Abstract

**Purpose of Review:**

Idiopathic normal-pressure hydrocephalus (iNPH) is characterized clinically by ventriculomegaly, abnormal gait, falls, incontinence, and cognitive decline. This article reviews recent advances in the pathophysiology of iNPH concerning sleep-disordered breathing (SDB) and glymphatic circulation during deep sleep.

**Recent Findings:**

The authors found iNPH frequently associated with obstructive sleep apnea (OSA). A critical factor in iNPH is intracranial venous hypertension delaying drainage of cerebrospinal fluid (CSF) into the cerebral venous sinuses. CSF-venous blood circulates in the jugular veins and finally drains into the heart. During SDB, repeated reflex attempts to breathe induce strong respiratory efforts against a closed glottis thereby increasing the negative intrathoracic pressure. This causes atrial distortion and decreases venous return to the heart resulting in retrograde intracranial venous hypertension. Additionally, repeated awakenings from OSA impede sleep-associated circulation of interstitial CSF into the glymphatic circulation contributing to hydrocephalus.

**Summary:**

Sleep has become a critical element in the cognitive changes of aging including iNPH.

## Introduction

Sleep is rapidly becoming the new frontier for the study of cognition, particularly in neurodegenerative dementias of aging [1•]. Normal sleep is critical for consolidation of memory and other cognitive functions; aging is associated with changes in sleep pattern that mediate age-related cognitive decline [2–4]. Sleep-disordered breathing (SDB), caused mainly by obstructive sleep apnea (OSA), affects daytime functioning by impairing memory, attention, and executive functions [5–7]. Numerous population-based studies [8–11, 12^•^] have shown that OSA is associated with up to 26% higher risk of cognitive impairment [12•], particularly in the elderly. Also, abnormal sleep has been associated with increased risk of Alzheimer disease (AD) [13–15].

Idiopathic normal-pressure hydrocephalus (iNPH) [16•] is a treatable form of dementia in the elderly with an average age of onset above 70 years. Approximately 700,000 persons might have iNPH in the USA in comparison with about 400,000 cases of multiple sclerosis. Unfortunately, although 60–80% of iNPH patients improve with shunt surgery, only a minority is diagnosed and treated. Patients with iNPH often have multiple vascular risk factors [17] including hypertension, diabetes, hyperlipidemia, smoking, hyperhomocysteinemia, coronary disease, stroke, and excessive body weight. Sleep studies have seldom been performed in patients affected by iNPH; therefore, SDB and OSA are rarely mentioned among the vascular risk factors [17]. This is due in part to the fact that both iNPH and OSA are largely under-diagnosed clinical conditions. However, with routine use of sleep questionnaires and polysomnography, we were able to demonstrate that SDB and iNPH are commonly associated, ranging in frequency from 65 to 90% [18••]. The latter observation serves as the basis for the present update.

### Clinical Manifestations of iNPH

The communicating hydrocephalus of iNPH is non-obstructive and characterized by enlargement of the cerebral ventricles with an Evans index greater than 0.30 (i.e., the ratio of the widest diameter of the frontal horns divided by the widest brain diameter on the same axial slice). Evans index values ≥ 0.33 indicate ventriculomegaly [19]. Other imaging features on magnetic resonance imaging (MRI) are a callosal angle ≥ 40° but < 90° [20] and narrowing of the sulci and subarachnoid spaces over the high convexity with enlarged Sylvian fissures, a feature called “disproportionately enlarged subarachnoid space hydrocephalus” (DESH) [21, 22]. Increased cerebrospinal fluid (CSF) stroke volume > 42 μL or pulsatile flow rates > 18 mL/min in the aqueduct of Sylvius on MRI, synchronized to the heartbeat for quantification, are considered good prognostic indicators for CSF shunting [23].

The clinical triad of iNPH includes cognitive loss, abnormal gait, and urinary incontinence. The cognitive problems include memory deficits and executive dysfunction predominantly frontal in nature. In their original description, Hakim and Adams [24•] mentioned, “psychomotor retardation … lack of impulsivity, expressed as apathy, disinterest, and lack of spontaneity.” Nevertheless, progressive gait difficulty and frequent falls may be the only manifestation of iNPH, without concurrent cognitive decline.

Gait imbalance resulting in frequent falls is a constant manifestation of iNPH [25]. Leg involvement is probably due to effects of ventriculomegaly on the tracts arising from the cortical representation of the lower limbs in the medial aspect of the primary motor cortex. Walking is described as wobbly, staggering, or drunken. Patients tend to fall without any obstacles, stumbling on minor floor irregularities, or while negotiating stairs and curbs. Gait initiation is slow and the feet appear to be “glued” to the floor or “magnetized.” Patients walk very slowly, with shuffling short steps. With the eyes closed, both the postural instability and the unsteadiness of gait increase markedly, indicating that both apraxia of gait and ataxia of leg movements in the vertical direction are present. Turning becomes precarious and is usually done by pivoting in one leg, i.e., the so-called compass sign [25]. At this point, patients usually require a cane or a walker to ambulate and are at very high risk of falls resulting in hip fractures and traumatic subdural hematomas. Hydrocephalus is often diagnosed in the emergency room during the evaluation of an elderly patient who sustained a fall. Falls among older adults are a major public health problem and by 2030 the number of fatalities is projected to reach 100,000 per year with an associated cost of $100 billion as a result of fractures, head injury, and other traumatic lesions [26].

The incontinence of sphincters is usually a manifestation of frontal dysfunction. According to Hakim and Adams [24•]: “… the patient being unaware of the contents of bladder and bowel is incapable of making any arrangement for the somewhat precipitate action of these organs.” Urinary incontinence is the second most common symptom of iNPH after gait problems and falls but it is often discounted as being the result of omnipresent prostate problems in aged men or an exacerbation of chronic bladder stress incontinence in elderly women. Urinary incontinence results in recurrent urinary tract infections and fatal septicemia. Frontal-type stool incontinence is less common but it also occurs in iNPH [25].

### Obstructive Sleep Apnea

OSA is the most prevalent form of SDB in adults [27•] affecting about 20 million Americans. SDB is an independent vascular risk factor—seldom diagnosed—that increases significantly the risk of cardiovascular complications [28, 29]. OSA was first reported by Gastaut and collaborators [30•] in a Pickwickian man with hypersomnia secondary to repeated airway obstructions by the tongue during sleep. Despite its prevalence, OSA remains grossly underdiagnosed: A recent systematic review of 24 population-based sleep studies using PSG among adults in the general population [31] found a worldwide increase in the prevalence of OSA. Severe OSA in older age groups reached an overall prevalence of 36% [32, 33]. A major risk factor for OSA is obesity, defined as a body mass index (BMI) > 30 kg/m^2^ [34]. OSA is a treatable cause of hypertension [35, 36], type 2 diabetes mellitus [37], and cardiovascular disease [28, 29], particularly atrial fibrillation [38–40], as well as pulmonary hypertension [41, 42], stroke, and transient ischemic attacks [43].

### Effects of OSA in Sleep

Early recordings of intracranial pressure during sleep demonstrated variations in CSF pressure during different sleep stages [44–46]. Table 1 provides a chronological summary of studies demonstrating elevation of intracranial pressure linked to the episodes of apnea in patients with OSA and concurrent iNPH. Several groups confirmed the presence of apnea-associated waves of increased CSF pressure (Lundberg B-waves) during rapid eye movement (REM) sleep [47–60].

**Table 1:** Early studies on the effects of obstructive sleep apnea on intracranial pressure, SaO_2_, and PaCO_2_

Author/year	Findings
Meyer et al., 1961 [47]	Pickwickian patient with papilledema, excessive sleepiness, hypoxemia, and hypercapnia; lumbar puncture showed elevation of CSF pressure to 480 mm H_2_O.
Lugaresi et al., 1978 [48]	45 OSA subjects: Arterial hypertension in 1/3 cases; all had transient hypoxemia and elevated PaCO_2_ with sleep apnea episodes; values worsened during REM sleep.
Iijima et al., 1979 [49]	OSA: Arterial blood gases showed transient hypoxemia and hypercapnia with apnea episodes.
Kaneda et al., 1983 [50]; Kuchiwaki et al., 1983 [51]	First described the association of OSA and NPH. ICP recording in patients with NPH showed increased ICP with presence of Lundberg B-waves with each apnea episode.
Kuchiwaki et al., 1984 [52]; 1988 [53]	17 patients with NPH and OSA showed elevation of CSF pressure during sleep apnea events. CSF shunting in 13 cases failed to improve the hypoxemia and hypercapnia observed with OSA. Authors suggested that OSA contributes to progression and worsening of hydrocephalus.
Sugita et al., 1985 [54]	3 patients with OSA: Marked increase of CSF pressure (50–750 mm H_2_O) measured at lumbar level following each episode of OSA/hypopnea. Longer apneas during REM sleep resulted in worse SaO_2_ decreases and higher increases of CSF pressure.
Jennum and Børgesen, 1989 [55]	6 OSA patients (none with NPH): Each apnea event increased ICP. ICP at rest was high (> 15 mmHg) and also in the morning (20·7 mmHg). While asleep, all patients developed apnea-associated elevated ICP.
Pasterkamp et al., 1989 [56]	1 patient with hydrocephalus treated with CSF shunt developed OSA years later: Rising intraventricular ICP up to 50 cm H_2_O occurred with each episode of apnea probably contributing to worsening syringomyelia.
McNamara et al., 1992 [57]	NPH symptoms worsened with nasal CPAP in 1 patient with NPH; treatment of NPH with VPS allowed use of CPAP with clinical improvement. CPAP and PEEP increase central venous pressure decreasing venous and CSF outflow, causing increased ICP.
Krauss et al., 1995 [58]	In 13 NPH patients, sleep apneas caused elevation of intraventricular ICP with Lundberg B-waves. Frequency of B-waves was higher during REM sleep and sleep stage 2.
Kristensen et al., 1998 [59]	Sleep-disordered breathing is very common in NPH: OSA was documented in 65% or 11/17 NPH patients. VPS failed to ameliorate sleep-disordered breathing in patients with NPH. OSA causes additional cognitive dysfunction in NPH patients.
Tsunoda et al., 2002 [60]	Using MRI, ventricular volume and intracranial CSF volume were increased in 17 patients with NPH; compared with controls, brain atrophy was also present in NPH patients.

*Abbreviations*: *CPAP*, continuous positive airway pressure; *CSF*, cerebrospinal fluid; *ICP*, intracranial pressure; *MRI*, magnetic resonance imaging; *NPH*, normal-pressure hydrocephalus; *OSA*, obstructive sleep apneas; *PaCO*_*2*_, arterial partial pressure of carbon dioxide; *PEEP*, positive end-expiratory pressure; *REM*, rapid eye movement; *SaO*_*2*_, arterial oxygen saturation; *VPS*, ventriculoperitoneal shunt

Apneas were preceded by a decrease in both arterial pressure and intracranial pressure and by increasing central venous pressure; this was reversed during the apnea along with lowering arterial SaO_2_ and increase SaCO_2_. At the termination of the apnea, a steep increase in arterial and intracranial pressures occurred. Intermittent CSF pressure values as high as 750 mm H_2_O were measured [54], well above the normal CSF pressure range (100–180 mm H_2_O or 8–15 mmHg), causing stress on the ventricular ependymal lining, the walls of the ventricles, and the surrounding brain structures.

It should be remembered that the CSF circulation is called “the third circulation” [61] because CSF production is linked to systemic arterial pressure and CSF reabsorption to the central venous pressure. This explains the interaction described above between respiration and intracranial pressure. CSF is produced by the choroid plexus inside the cerebral ventricles from arterial blood at rates dependent on systolic blood pressure.

After circulating in the ventricles and around the cerebral and spinal subarachnoid space, CSF is reabsorbed into the intracranial venous circulation at the level of the Pacchionian granulations that protrude into the lumen of the cerebral venous sinuses, particularly into the superior longitudinal sinus. The CSF-venous blood admixture drains into the internal jugular veins, then the brachiocephalic or innominate veins, the superior vena cava, and finally into the right atrium and the heart.

### SDB-Induced Intracranial Venous Hypertension

**Fig. 1:**
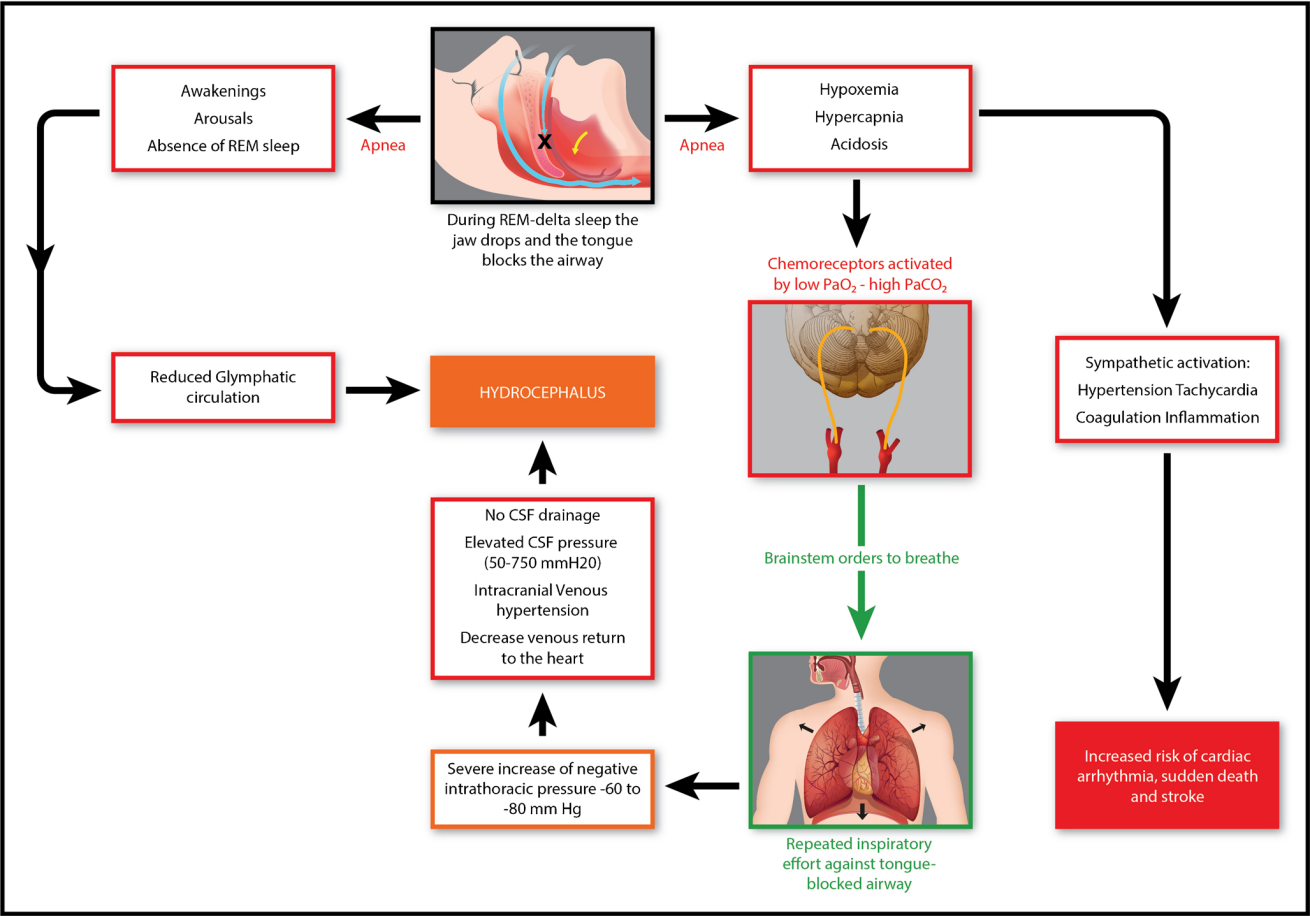
In subjects with SDB, sleep apneas occur more frequently during deep sleep stages, i.e., REM sleep and delta sleep. Muscle paralyses typical of deep sleep stages lead to opening of the mouth and relaxation of tongue muscles (top central image). The resulting apnea (right arrow) causes hypoxemia, hypercapnia, and acidosis that activate arterial chemoreceptors. In turn, this activation is answered by respiratory brainstem nuclei, which order the inspiratory muscles to inhale (downward green arrow). Repeated inspiratory movements against the closed airway cause severe increase of negative intrathoracic pressure (− 60 to − 80 mmHg), decrease of venous return to the heart, intracranial venous hypertension, absence of CSF drainage into the venous sinuses, and hydrocephalus. Sleep apneas also cause fragmentation of the sleep architecture (top central image, left arrow) with arousals and awakening, as well as lack of REM sleep. Absence of REM sleep is accompanied by decreased glymphatic circulation of CSF, which contributes to hydrocephalus. The metabolic consequences of the sleep apnea syndrome are illustrated on the right side of the diagram and result from sympathetic activation, with tachycardia, hypertension, activation of coagulation mechanisms, and systemic inflammatory responses that increase the risk of cardiac arrhythmias, sudden death, and stroke

Figure 1 summarizes the main pathophysiological cardiovascular mechanisms linking sleep apneas and hydrocephalus. Fragmentation of the sleep architecture induced by apneas reduces glymphatic circulation and further contributes to iNPH.

#### Cardiovascular Mechanisms

In 2008, Williams [62] proposed that the main pathogenic mechanism in hydrocephalus is *intracranial venous hypertension* whereby the increased intracranial venous pressure hinders CSF absorption through the arachnoid villae in the dural sinuses resulting in excessive accumulation of intracranial CSF. A number of studies have confirmed the postulated venous hypertension hypothesis [63–66]. The cardiovascular and metabolic mechanisms resulting from OSA capable of inducing intracranial venous hypertension are summarized in Table 2.

**Table 2:** Pathophysiological mechanisms relevant to iNPH induced by apneas during sleep

• Recurrent hypoxemia (low PaO_2_) hypercapnia (elevated PaCO_2_) and respiratory acidosis (low blood pH) • Activation of carotid and aortic chemoreceptors	
• Stimulation of rostral pontine respiratory neurons	
• Firing of solitary tract nucleus neurons, dorsal medullary respiratory group, and ventral group nucleus ambiguus • Repeated reflex contractions of respiratory chest and abdominal muscles	
• Thoracoabdominal excursions greatly increase negative intrathoracic pressure	
• Superior vena cava and intracranial venous hypertension in the dural sinuses	
Decreased CSF absorption through Pacchionian granulations (arachnoid villae)	
Tachycardia from atrial Bainbridge reflex	
• Sympathetic (adrenergic) outburst	
Arterial hypertension	
Baroreceptor reflex activation	
Peripheral vasoconstriction	
Hyperglycemia	
Hypercoagulability	
• Inflammation from recirculation of hypoxic-acidotic blood	
C-reactive protein (CRP)	
Nuclear factor-κΒ (NF-κΒ)	
Hyperhomocysteinemia	
Interleukin-6 (IL-6)	
Tumor necrosis factor alpha (TNF-α)	

Obstructed breathing during sleep causes marked increase of the negative intrathoracic pressure and hypoxemia. The interruption of ventilation in OSA is usually due to relaxation of pharyngeal and tongue muscles causing airway obstruction at the pharynx [67–69]. This occurs most often during the global loss of muscle tone and relaxation of muscles typical of slow-wave NREM and REM sleep. The extrinsic muscles of the tongue (genioglossus, geniohyoid) insert on the mandible’s inner surface. Slow-wave and REM sleep cause the jaw to drop and the tongue to fall back blocking the airway. Concurrent contracture of pharyngeal constrictor muscles further closes the airway. Supine and open-mouth sleepers from chronic obstruction of nasal passages tend to have more apneas than those who sleep with a closed mouth or in lateral decubitus.

Hypoxemia occurs as a result of the interruption of respiration (apnea or hypopnea), lowering of PaO_2_ with concomitant hypercapnia from CO_2_ retention, elevation of PaCO_2_, and respiratory acidosis [69, 70]. Patients with severe OSA (Apnea-Hypopnea Index, AHI ≥ 30/h) may exhibit > 60 apneas per hour of sleep (i.e., > 1 apnea/min) with severe metabolic consequences [66–71].

The main stimulus for respiratory inspiration is the activation by the low PaO_2_ of carotid and aortic body chemoreceptors and brainstem respiratory chemoreceptors responding to increased PaCO_2_ and lower blood pH as a result of respiratory acidosis. The net result of the stimulation of chemoreceptors is the activation of rostral pontine respiratory neurons that in turn stimulate firing of neurons in the solitary tract nucleus and the dorsal medullary respiratory group and ventral group in the nucleus ambiguus [72, 73].

Inspiratory impulses are carried by the phrenic and intercostal nerves, stimulating motor activity of the diaphragm, respiratory inspiratory muscles, and pharyngeal dilator muscles. The contraction of diaphragm and intercostal muscles results in expansion of the thoracic cavity. The inspiratory effort concludes when pulmonary stretch afferences stimulate the pontine apneustic center that in turn inhibits medullary inspiratory neurons [72, 73].

These reflex attempts to breathe cause intermittent, often violent, and strenuous respiratory efforts that involve chest and abdominal musculature trying to overcome the obstruction of the airway to restore airflow. This is the Mueller maneuver (the opposite of Valsalva’s) that generates severely negative intrathoracic pressure (− 60 to − 80 mmHg) [68–71]. The resulting atrial distortion is a major risk factor for atrial fibrillation [67–69, 74].

Suffocation is the end result of these unsuccessful respiratory efforts to improve oxygenation producing an acute stress reaction with microarousals, awakening and disruption of sleep due to the sympathetic (adrenergic) outburst resulting, among other effects, in arterial hypertension, peripheral vasoconstriction, hyperglycemia, and hypercoagulability [75–77]. OSA is the commonest cause of drug-resistant secondary hypertension [78].

Subsequent reoxygenation and recirculation of hypoxic and acidotic blood worsens hypoxemia and results in generation of reactive oxygen species (ROS), C-reactive protein (CRP), homocysteine (Hcy), and other inflammatory factors [79, 80]. The recirculation of anoxic and acidotic blood has major inflammatory effects and explains the increased risk of coronary artery disease, stroke, and small-vessel cerebrovascular disease found in patients with OSA [66, 81].

The apnea-induced negative intrathoracic pressure opposes the venous return to the heart resulting in elevation of venous pressure in the superior vena cava and internal jugular vein system. The authors propose that the end result of untreated SDB is intracranial venous hypertension. This leads to reduction of the normal drainage of CSF into the superior sagittal sinus and other dural sinuses eventually causing progressive accumulation of CSF inside the ventricles, ventriculomegaly, and symptomatic NPH.

The statistically significant association of OSA with NPH is relatively novel [18^••^] but the physiopathological vascular and metabolic mechanisms have been amply supported in the literature [66–71, 74–80]. For instance, pulmonary hypertension is a well-known effect of OSA [82] and positive end-expiratory pressure (PEEP) [83–85] can increase central venous pressure, decreasing outflow and elevating intracranial CSF pressure. Frydrychowski et al. [86] showed that acute increases in jugular vein pressure induce elevation of intracranial CSF pressure. Lee et al. [87] postulated a similar mechanism to explain the induction of papilledema in obese subjects with pseudotumor cerebri [87] particularly in the presence of OSA [88].

### Alterations of Glymphatic Circulation in iNPH

The glymphatic (glial-lymphatic) pathway of the brain [89••, 90••] directs the flow of CSF along arterial perivascular spaces into the brain interstitial spaces, facilitated by aquaporin 4 (AQP4) water channels [91, 92]. CSF circulation in the glymphatic system in rodents increases twofold during deep delta sleep and correlates with increase in AQP4 [90••]. The flow of CSF reaches the venous perivascular and perineuronal spaces, ultimately draining into meningeal and cervical lymphatic vessels. The overall function of the glymphatic system is the clearance of the brain parenchyma from metabolic leftovers and interstitial solutes including beta amyloid. Decreased brain clearance may contribute to the development of neurodegenerative diseases [91].

In patients with iNPH, Hasan-Olive et al. [93] performed electron microscopy studies in cortical brain biopsies and demonstrated reduced density of AQP4 water channels in astrocytic end-foot membranes along cortical microvessels. Ringstad et al. [94••] utilized intrathecal gadobutrol MRI in subjects with iNPH and observed delayed clearance of the CSF tracer due to resistance to glymphatic flow. Parenchymal glymphatic enhancement peaked overnight as an effect of sleep but glymphatic clearance was clearly decreased in iNPH patients. According to Ringstad et al. [94••], the decreased glymphatic clearance explains the delayed periarterial enhancement of glymphatic flow and the reflux of gadobutrol into the lateral ventricles typical of iNPH. Reduced glymphatic function probably related to abnormal sleep resulting from SDB may be instrumental in the pathogenesis of iNPH.

## Conclusions

The pathogenesis of iNPH has remained enigmatic during the half century since its description. However, the association of SDB and iNPH was well documented and studied in the years that followed the first accounts of these two conditions in 1965 [24•, 30•]. The pathophysiological interactions between respiration and CSF circulation have been solidly established since then [66]. However, those earlier observations were essentially forgotten until we rediscovered the interaction by the systematic assessment of sleep in all patients evaluated at our Memory Disorders and Dementia Clinic [18••]. Recent and future discoveries of sleep physiology relevant to cognitive disorders of aging should continue to increase our understanding of iNPH perhaps opening new venues for treatment and prevention.
